# Phytochemical Composition and In Vitro Biological Activity of *Iris* spp. (Iridaceae): A New Source of Bioactive Constituents for the Inhibition of Oral Bacterial Biofilms

**DOI:** 10.3390/antibiotics9070403

**Published:** 2020-07-11

**Authors:** Lan Hoang, František Beneš, Marie Fenclová, Olga Kronusová, Viviana Švarcová, Kateřina Řehořová, Eva Baldassarre Švecová, Miroslav Vosátka, Jana Hajšlová, Petr Kaštánek, Jitka Viktorová, Tomáš Ruml

**Affiliations:** 1 Department of Biochemistry and Microbiology UCT Prague, Faculty of Food and Biochemical Technology, Technická 3, 166 28 Prague, Czech Republic; hoangl@vscht.cz (L.H.); kronusova@ecofuel.cz (O.K.); fuchsovv@vscht.cz (V.Š.); rehorova@vscht.cz (K.Ř.); rumlt@vscht.cz (T.R.); 2 Department of Food Analysis and Nutrition UCT Prague, Faculty of Food and Biochemical Technology, Technická 3, 166 28 Prague, Czech Republic; benesfr@vscht.cz (F.B.); fenclovm@vscht.cz (M.F.); hajslovj@vscht.cz (J.H.); 3 EcoFuel Laboratories Ltd., Ocelářská 9, 190 00 Praha, Czech Republic; kastanek@ecofuel.cz; 4 Institute of Botany of the Czech Academy of Sciences, Zámek 1, 252 43 Průhonice, Czech Republic; eva.svecova@ibot.cas.cz (E.B.Š.); miroslav.vosatka@ibot.cas.cz (M.V.)

**Keywords:** biofilm, dental plaque, quorum sensing, microbial resistance

## Abstract

The inhibition and eradication of oral biofilms is increasingly focused on the use of plant extracts as mouthwashes and toothpastes adjuvants. Here, we report on the chemical composition and the antibiofilm activity of 15 methanolic extracts of *Iris* species against both mono-(*Pseudomonas aeruginosa*, *Staphylococcus aureus*) and multi-species oral biofilms (*Streptococcus gordonii, Veillonella parvula, Fusobacterium nucleatum* subsp. *nucleatum*, and *Actinomyces naeslundii*). The phytochemical profiles of *Iris pallida* s.l., *Iris versicolor* L., *Iris lactea* Pall., *Iris carthaliniae* Fomin, and *Iris germanica* were determined by ultra-high performance liquid chromatography-high-resolution tandem mass spectroscopy (UHPLC-HRMS/MS) analysis, and a total of 180 compounds were identified among *Iris* species with (iso)flavonoid dominancy. *I. pallida, I. versicolor,* and *I. germanica* inhibited both the quorum sensing and adhesion during biofilm formation in a concentration-dependent manner. However, the extracts were less active against maturated biofilms. Of the five tested species, *Iris pallida* s.l. was the most effective at both inhibiting biofilm formation and disrupting existing biofilms, and the leaf extract exhibited the strongest inhibitory effect compared to the root and rhizome extracts. The cytotoxicity of the extracts was excluded in human fibroblasts. The inhibition of bacterial adhesion significantly correlated with myristic acid content, and quorum sensing inhibition correlated with the 7-β-hydroxystigmast-4-en-3-one content. These findings could be useful for establishing an effective tool for the control of oral biofilms and thus dental diseases.

## 1. Introduction

Bacterial biofilms, communities of microorganisms in a self-produced extracellular polymeric substance matrix, cause more than 60% of human microbial infections [1]. Changes in gene expression and the activation of numerous extracellular communication pathways during biofilm formation often lead to an increase in pathogenicity and overall virulence activity. Startlingly, the antimicrobial resistance of a biofilm can be up to a thousandfold higher than that of free planktonic biota [2].

Quorum sensing (QS) is known as a cell–cell communication pathway that initiates and regulates various physiological activities such as biofilm formation, bioluminescence, and virulence production. Both Gram-positive and Gram-negative bacteria use QS for communication, but they produce distinct signal molecules (autoinducers): N-acyl homoserine lacton (AHL) molecules (autoinducer-1, AI-1) are mainly used by Gram-negative bacteria, while Gram-positive bacteria predominantly use modified oligopeptides (autoinducer peptides, AIP or QS peptides). Another type of signal molecules is autoinducer-2 (AI-2), which are derived from boron-furan and found in both Gram-negative and Gram-positive bacteria. In addition, there is also a fourth class of miscellaneous QS molecules. These QS molecules are not only responsible for inter-kingdom communication, but are possibly also involved in the direct or indirect cross-talk between microorganisms and their environment. Thus, the idea of using antimicrobials, which interfere with the microorganism’s QS mechanism, has been a novel antipathogenic method for inhibiting biofilm formation with minimal side effects that is also non-toxic to the host [3].

An oral biofilm contains hundreds of different oral bacteria that may cause serious diseases within the oral cavity. Furthermore, the virulence in response to drastic changes in the biofilm microenvironment can be spread systemically and may induce significant infections in other organs [4]. The presence of *Staphylococcus aureus* in a supra- and subgingival biofilm can induce periodontitis [5], while *Pseudomonas aeruginosa* from a subgingival biofilm may be responsible for a more aggressive form of periodontitis [6]. Dental plaque, consisting of both Gram-positive and Gram-negative bacteria such as *Streptococcus gordonii* and *Fusobacterium nucleatum*, is able to adhere to tooth surfaces, proliferate, and produce lactic acid, causing the demineralization of dental enamel and dentine.

Limitations of conventional antibiotic therapy as well as increasing drug resistance have led to the urgent need for alternative approaches to deal with oral biofilm-related infections. In this context, the use of biologically active plant extracts as antibiotic adjuvants has been of great interest over the last few decades for exhibiting broad biological activities [7]. E.g., various extracts of *Vitis vinifera* have been shown to eradicate oral microorganisms via various mechanisms, including enzyme inhibition, cell wall disruption, and QS inhibition [8,9]. Other studies revealed a high antimicrobial efficacy of *Coffea canephora* [10,11], green tea [12], and *Chesneya nubigena (D. Don) Ali* [13]. Despite these pioneering works and promising results, the antimicrobial potential of a wide range of plants remains to be explored.

*Iris* spp. is the largest genus of the Iridaceae family and is one of the most important genera of flowering plants, with a rich diversity growing in the territories of Eurasia and North America. The species of this genus have been used in traditional medicine. Due to its rich diversity, the genus *Iris* represents a reservoir of valuable species not only for cultivation purposes, but also as a source of biologically active substances. A broad range of secondary metabolites isolated from *Iris* spp. have exhibited numerous biological activities such as antibacterial, antioxidant, anti-inflammatory, anti-cancer, and immuno-modulatory [14,15,16,17,18,19,20,21,22]. However, a literature review revealed that there is very limited information about the anti-biofilm activity of *Iris* plants, especially against oral biofilms.

Here, we report on the phytochemical composition and the *in vitro* effect of extracts from five *Iris* spp. on the adherence and disruption of oral microbial biofilms. Moreover, their mechanisms of action against the virulence factors of oral bacteria are described. Given that these bacteria express distinct QS autoinducers that play important roles in the development of virulence factors, we also investigated the effect of *Iris* spp. on the QS communication pathway and provide an additional theoretical basis for its application.

## 2. Materials and Methods

### 2.1. Plant Materials and Preparation of Extracts

The different tissues of *Iris* plants (leaves, roots, rhizomes) used in this study were collected from the field collection in the Botanical garden of the Institute of Botany, Czech Republic (July 2018). Taxonomic identification of the plant materials was confirmed by Dr. Z. Caspers, a herbarium specialist from the Botanical garden.

The plant materials were washed in distilled water and cut into small pieces. After cleaning, the parts were air-dried at room temperature for four days to remove the residual moisture and ground into a fine powder using a laboratory mill. To produce methanol extracts, 1 g of fine plant powder was macerated with 15 mL of 80% methanol at room temperature for 15 h. After that, the extracts (66.7 mg/mL) were filtered using filter paper and stored at –20 °C before their use.

### 2.2. Phytochemical Analysis: Ultra-High-Performance Liquid Chromatography Coupled with High-Resolution Tandem Mass Spectrometry (UHPLC–HRMS/MS)

For the purpose of phytochemical profiling, an internal database of secondary metabolites reported in *Iris* spp. plants was created based on a scientific literature search [23]. Then, those predicted compounds were screened in a targeted manner in the crude extracts using UHPLC-HRMS/MS analysis, as previously described by [24] with some modifications. Chromatographic separation was achieved using a 150 × 2.1 mm i.d., 1.7 μm Acquity UPLC^®^ BEH C_18_ column (Waters, Milford, MA, USA) in a chromatographic Agilent 1290 Infinity LC System (Agilent Technologies, Santa Clara, CA, USA). The mobile phases consisted of water/acetonitrile (95:5, v/v) (A) and 2-propanol/acetonitrile/water (75:20:5, v/v/v) (B), both containing ammonium acetate (5 mM) and acetic acid (0.1%). The gradient was as follows: 0–0.5 min, flow 0.3 mL/min, 100% **A**; 0.5–4 min, flow 0.3 mL/min, 100–35% **A**; 4–8 min, flow 0.2 mL/min, 35–22.5% **A**; 8–13 min, flow 0.2 mL/min, 22.5–0% **A**; 13–18 min, flow 0.35 mL/min, 0% **A**. Then, the column was equilibrated for 2 min under the initial conditions. The injection volume was 1.0 μL, and the column temperature was maintained at 60 °C.

The Agilent 6560 quadrupole–time of flight mass spectrometer (Q-TOF) (Agilent Technologies, Santa Clara, CA, USA) was operated in Q-TOF Auto MS/MS acquisition mode. The specific parameters for the mass spectrometer were as follows: electrospray ionization both in positive and negative polarity (separate injections of the samples); drying gas flow rate 12 L/min; drying gas temperature 280 °C; sheath gas flow rate 12 L/min; sheath gas temperature 350 °C; nozzle voltage 400 V; capillary voltage 3500 V; nebulizer 40 psig. In the Auto MS/MS mode, the following parameters were used: mass range 100–1000 m/z (both in MS and MS/MS); acquisition rate 3 spectra/s (MS) and 12 spectra/s (MS/MS); collision energy 20 eV. The predicted compounds of *Iris* spp. were detected and tentatively identified based on the exact masses (*m/z*) of their precursor ions, their isotopic patterns and where possible, the agreement of recorded MS/MS spectra with online mass spectral libraries (such as ‘METLIN’, ‘mzCloud’), or the scientific literature. For some of the detected compounds, several chromatographic peaks meeting the HRMS criteria were observed, probably indicating the presence of structural isomers.

### 2.3. Antimicrobial Activity

The antimicrobial activity of the extracts was tested against eight pathogenic microorganisms: *Pseudomonas aeruginosa* (CCM, 3955), *Staphylococcus aureus* (ATCC, 25923), *Salmonella enterica* (CCM, 4420), *Candida albicans* (DBM, 2186), *Streptococcus gordonii* (DSMZ, 6777), *Veillonella parvula* (DSMZ, 2008), *Fusobacterium nucleatum* subsp. *nucleatum* (DSMZ, 15643), and *Actinomyces naeslundii* (DSMZ, 43013). The selected strains were according to the EUCAST (European Committee on Antimicrobial Susceptibility Testing) antibiotic-sensitive, which was verified by cefotaxime and penicillin sensitivity. IC_50_ of penicillin [mg/L] was as follows: 0.0059 ± 0.0001 for *S. aureus*; IC_50_ of cefotaxime [mg/L] was as follows: 0.55 ± 0.05 for *P. aeruginosa*, 0.95 ± 0.05 for *C. albicans*, 0.058 ± 0.003 for *S. gordonii*, 0.0047 ± 0.0004 for *V. parvula*, and 0.017 ± 0.0004 for *F. nucleatum* and 0.022 ± 0.001 for *A. naeslundii.*

Susceptibility tests of the target microorganisms, both Gram-positive and Gram-negative bacterial strains, and yeast, were carried out using the standard broth microdilution method, in 96-well plates as described previously [25]. The tested bacteria and yeasts were grown overnight in Brain Heart Infusion Broth (BHI, Sigma-Aldrich, St. Louis, MO, USA) and Malt extract broth (ME broth, Oxoid, Hampshire, UK), respectively. Resulting suspensions were adjusted to a turbidity of 0.5 McFarland. The extracts were 100× diluted with the suspensions and then binary diluted with the same suspension. These diluted extracts were added to 96-well plates providing concentrations of the extracts ranging from 0.7 up to 666.7 mg/L. All experiments were conducted with a maximum of 1% (v/v) methanol in solution. The suspension of microorganisms without the tested compounds served as a positive control. Bacterial and yeast cultures were incubated for 24 h at 120 rpm and 37 and 28 °C, respectively, and the absorbance was recorded at 500 nm using the SpectraMax i3x Multi-Mode Detection Platform (Molecular Devices, San Jos Tibco Software Inc., San Jose, CA, USA).

### 2.4. Anti-Biofilm Activity

The activity of the *Iris* extracts on mono- and multi-species bacterial biofilms was tested using *Staphylococcus aureus* (ATCC, 25923), *Pseudomonas aeruginosa* (CCM, 3955), and dental plaque, which consisted of four oral bacterial strains: *Streptococcus gordonii* (DSMZ, 6777), *Veillonella parvula* (DSMZ, 2008), *Fusobacterium nucleatum* subsp. nucleatum (DSMZ, 15643), and *Actinomyces naeslundii* (DSMZ, 43013). *S. aureus* and *P. aeruginosa* were incubated in BHI broth medium at 37 °C aerobically, while all the dental plaque strains were propagated anaerobically using an anaerobic jar (model HP0031A, Thermo Fisher Scientific, MA, USA). An anaerobic atmosphere of 80% N_2_, 10% CO_2_, and 10% H_2_ was obtained with an Oxoid™ AnaeroGen™ 3.5L Sachet with Thermo Scientific™ Resazurin Anaerobic Indicator BR0055 (Thermo Fisher Scientific, MA USA).

For single-species biofilms, the method described by [26] was used. For the mixed biofilm, overnight cultures adjusted to 0.5 McFarland turbidity of all species: *S. gordonii*, *V. parvula*, *F. nucleatum,* and *A. naeslundii* were mixed in the same ratio (1:1:1:1, v/v). After that, 100 μL was split into each well and incubated for 48 h. For testing the anti-adhesion activity, a resazurin assay was employed to evaluate the viability of attached cells immediately after 24 h of incubation in the presence of the tested extracts at 37 °C and washing with Phosphate Buffered Saline (PBS) three times (pH 7.4). In the eradication of mature biofilms, the medium was discarded after the adherence incubation period of all strains, and fresh BHI broth medium was replaced every 24 h for 7 days of incubation under anaerobic conditions in order to allow biofilm maturation. After that, the extracts were added in a concentration range of 0.7–666.7 mg/L and incubated for another 24 h. After the final washing of the biofilm, the viability was determined by resazurin assay [27]. The resazurin (Sigma-Aldrich) in PBS (0.03 mg/L) was incubated with the cells for 2 hours at 37 °C avoiding light exposure. The production of resorufin was quantified by measuring fluorescence (560/590 nm, ex./em.) using the SpectraMax i3x Multi-Mode Detection Platform (Molecular Devices, USA). The viability of cells was calculated relative to the viability of cells in the absence of the tested samples.

### 2.5. Anti-Quorum Sensing Activity

The production of bioluminescence by two commercial (The American Type Culture Collection, ATCC) strains of *Vibrio campbellii*—BAA1118 and BAA1119 in the presence or absence of tested extracts was determined for the evaluation of anti-QS according to the previous protocol [26]. Autoinducer Bioassay (AB-A) medium, consisting of NaCl (17.5 g/L), MgSO_4_ (12.3 g/L), casamino acids (2 g/L), 10 mM potassium phosphate (pH 7.0), 1 mM L-arginine, and glycerol (10 mL/L) was used for inoculating these two strains and all anti-QS experiments. The 0.2 McFarland overnight culture in AB-A medium was split into each well with the binary dilution of *Iris* extracts (0.7 mg/L–666.7 mg/L). At first, the viability of *V. campbellii* was checked by resazurin assay for setting up the experiment at non-toxic concentrations of the tested samples. IC_10_ was chosen for the anti-QS assay. The extracts were applied at the IC_10_ concentration and further binary diluted with the cell suspension. Then, luminescence was recorded for 16 h with a measurement step of 20 min using a microplate reader set up at 30 °C; integration time of 10,000 ms; and shaking for 60 s prior to measurement. After the measurements, the QS IC_50_ was calculated based on the sum of luminescence recorded by a microplate reader (SpectraMax i3 Multi-Mode Detection Platform, Molecular Devices, UK).

### 2.6. Cytotoxicity Assay

The extracts were evaluated for their *in vitro* cytotoxicity using human fibroblasts (MRC-5, Sigma-Aldrich, USA) obtained from ATCC (USA). The cell line was grown in Eagle’s Minimum Essential Medium (EMEM) culture medium containing 10% fetal bovine serum (FBS) and 1% antibiotic mixture (penicillin, 100 IU/mL and streptomycin, 100 g/mL) at 37 °C in a 5% CO_2_ humidified incubator. The cells were counted with a Cellometer Auto T4 (Nexcelom Bioscience, Lawrence, MA), seeded (1 × 10^5^ cells/mL) in a 96-well plate and incubated for 24 h. Then, the cell culture medium was discarded from each well, and the tested extracts were added to assess the effect on cytotoxicity. After 72 h of incubation, a standard resazurin assay [28] was performed to determine the cell viability. The results were expressed as a percentage of viable cells compared to the control (taken as 100%).

### 2.7. Data Processing and Statistical Analysis

Unless otherwise stated, results are presented as an average of triplicates with the appropriate standard error of the mean (SEM). The relative activity was evaluated as a percentage according to the formula:

$$RA\;\left(\%\right)=100\frac{\left(\mathrm{slope}\;\mathrm{of}\;\mathrm{sample}-\mathrm{average}\;\mathrm{slope}\;\mathrm{of}\;\mathrm{PC}\right)}{\left(\mathrm{average}\;\mathrm{slope}\;\mathrm{of}\;\mathrm{NC}-\mathrm{average}\;\mathrm{slope}\;\mathrm{of}\;\mathrm{PC}\right)}.$$

Values of IC_50_ were determined using an online tool freely provided by AAT Bioquest – IC_50_ Calculator.

The results were subjected to one-way analysis of variance (ANOVA) followed by Duncan’s post hoc test (*p* < 0.05) to show significant differences between the means of treated and untreated groups as identified in each assay. Statistica software version 12 (Tibco Software Inc., Tulse, OK, USA) was employed in the ANOVA analysis.

The correlation coefficients were calculated using the automatic function “CORREL” in Microsoft® Office Excel according to [24]. The following variables were used as the matrix: (I) IC_50_ for specific biological activity and (II) the peak areas of compounds detected and tentatively identified by targeted UHPLC–HRMS/MS screening. The significance of the correlation coefficient was evaluated using a comparison of coefficients and the critical values (α = 0.05), which were determined using the degrees of freedom (df = n−2).

## 3. Results

### 3.1. Phytochemical Analysis of Plant Extracts 

The profile of phytochemicals detected using the UHPLC-HRMS/MS method in a methanol extract of leaves, roots, and rhizomes from 5 different species of *Iris* spp. is presented in Table 1 and Table A1. As seen in Table 1, more than 50 compounds were found in each extract, with (iso)flavonoids predominating. The highest number of (iso)flavonoids was detected in *I. pallida* leaves and roots, which consisted of 35 and 38 (iso)flavonoids, respectively, while less than half of them were detected in the rhizomes of this species. The lowest deviation in the composition was observed for steroids and fatty acids; in contrast, quinones and flavonoids exhibited variation in their presence within the species. A total of 180 individual compounds were detected and tentatively identified in the samples as a result of the screening analysis. In the extracts, 25 compounds were detected in only 1 sample, while 33 compounds were present in more than half of the extracts. The compound’s name, molecular formula, experimentally obtained neutral exact mass, retention time (t_R_, min), and presence in 3 different parts of five *Iris* spp. are summarized in Table A1.

### 3.2. Antimicrobial Activity

For the determination of antimicrobial activity of the *Iris* extracts, both Gram-positive (*S. aureus*, *B. cereus*, *S. gordonii*, *V. parvula*, *A. naeslundii*) and Gram-negative (*P. aeruginosa*, *S. enterica*, *F. nucleatum*) bacteria and yeast (*C. albicans*) were tested. However, no antibacterial or antifungal activity was observed, even at the highest tested concentration of 666.7 mg/L (data not shown). Therefore, this concentration was chosen as the sub-minimum inhibitory concentration (sub-MIC) and used in further experiments.

### 3.3. Anti-Biofilm Activity

The sub-MIC of *Iris* methanol extracts significantly inhibited the adhesion of Gram-positive (*S. aureus*) and Gram-negative (*P. aeruginosa*) bacteria as well as the dental plaque multispecies biofilm in a concentration-dependent manner (Table 2, Supplementary Table S2). Specifically, activity suppressing biofilm formation was observed more in leaf, root, and rhizome extracts (*I. pallida*, *I. versicolor*), with weaker activity in extracts from leaves and rhizomes (*I. germanica*) and roots (*I. lactea*). Furthermore, the IC_50_ for the multi-species biofilm was significantly higher than those for the mono-species one. Compared to mono-species biofilms, higher concentrations were required to prevent the formation of the multi-species biofilm. Out of all five species, only all the extracts of *I. pallida* and *I. versicolor* reduced multi-species cell adhesion by more than 50%.

**Figure 1:**
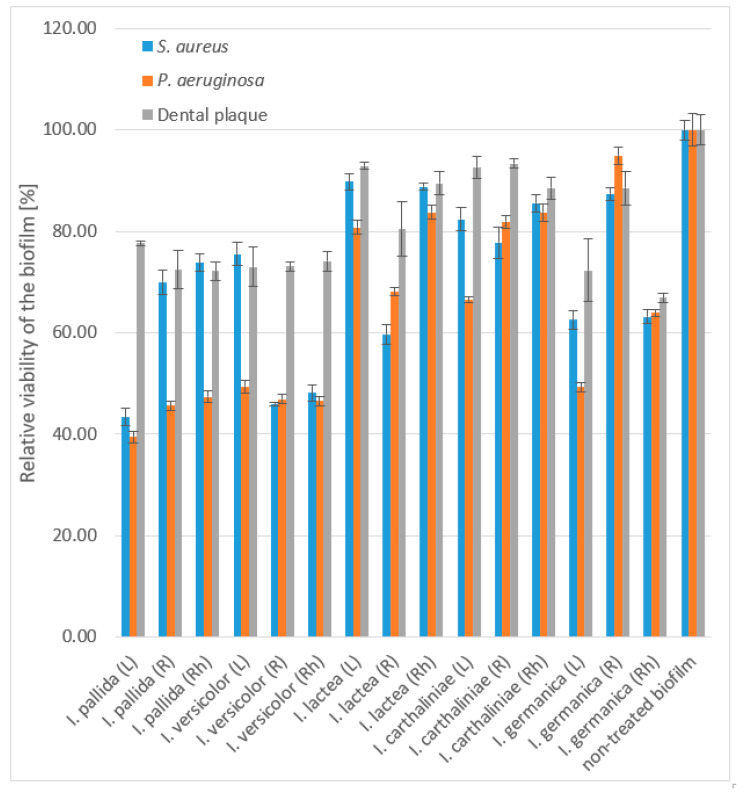
Disruption of mature biofilm by methanol extracts of Iris spp. at concentration of 666.7 mg/L. Data are presented as average of 3 repetitions with SEM. For the statistical analysis, see Supplementary Table S1.

In general, the methanol extracts of *I. pallida* and *I. versicolor* exhibited strong eradication effects on all tested biofilms in both stages: cell adhesion and disruption of a maturated biofilm, with a higher activity against Gram-negative bacteria (*P. aeruginosa*, roots, rhizomes). The leaf extract of *I. pallida* demonstrated the strongest inhibitory effect, as it disrupted the mature *P. aeruginosa* biofilm with the IC_50_ 0.29 ± 0.01 g/L followed by *I. versicolor* root and rhizome extracts. Of the other three extracts, only the leaf extract of *I. germanica* disrupted the matured biofilm observed for *P. aeruginosa*. This activity was not observed in other extracts. Furthermore, none of the *Iris* extracts at the highest tested concentration were able to significantly eradicate the multispecies biofilm of *S. gordonii, V. parvula, F. nucleatum,* and *A. naeslundii* (Figure 1, Supplementary Table S1).

### 3.4. Cell-To-Cell Communication Inhibition Assay in V. campbellii

In this study, we investigated the QS inhibitory potential of 15 *Iris* methanolic extracts against the QS-dependent phenotypic production of luminescence in mutant sensor strains of *V. campbellii* responding either only to (1) AI-1 autoinducer (BAA1118) or (2) AI-2 autoinducer (BAA1119). To avoid false positive results in the QS inhibition experiment, the concentration of 666.67 mg/L was determined as non-toxic to the tested strains. Thus, the reduced bioluminescence production resulted from an inhibition of cell–cell communication rather than an inhibition of cell growth. In general, only 6 extracts exhibited an inhibition of homoserine lactones-mediated luminescence production in *V. campbellii* BAA1118, responding to autoinducer 1 (AI-1), while AI-2-mediated communication was only inhibited by three extracts (Table 3, Supplementary Table S3). *I. lactea* and *I. carthaliniae* had no effect on QS at all. The *I. pallida* leaf extract inhibited communication based on both AI-1 and AI-2 systems similarly to the root and rhizome extracts of *I. versicolor*. Although they inhibited the cell-to-cell communication system based on boron compounds (AI-2) implemented by many Gram-negative and Gram-positive bacteria, their activities were significantly higher against AI-1. An inhibition of intercellular bacterial communication based on the AI-1 autoinducer was observed in *I. pallida* (leaves, roots), *I. versicolor* (all tissues), and *I. germanica* (leaves). No anti-QS activity was found in other extracts.

### 3.5. In Vitro Cytotoxicity 

It is necessary to determine the possible toxic effects of antimicrobial agents on human cells to confirm the safety of antimicrobial agents at their effective concentrations intended for application within the oral cavity. The cytotoxic effect of 15 samples on the human fibroblastic cell lines was evaluated by resazurin assay after 72 h of exposure. As shown in Table 4, no toxicity was observed for fibroblasts (MRC cell line) at the highest tested concentration (670 mg/L), except for the rhizome extracts from *I. versicolor* and *I. carthaliniae*. 

### 3.6. Correlation of Biological Activities and Extract Composition

To determine the relationship between the biological activity response in the particular test examined and the amount of the compounds present in the samples, the correlation of the biological activity results with the HRMS/MS responses of detected compounds in each extract was calculated. When plotting the results for all 15 of the extracts, the correlation coefficient (R^2^) was determined and assessed. The ability of the extracts to inhibit dental plaque adhesion significantly correlated with the content of myristic acid and germanaism B. Three compounds from different chemical groups: 7-β-hydroxystigmast-4-en-3-one(steroid), amorphene/α-muurolene/β-gurjuenene/γ-elemene (terpenoid), and isomangiferin/mangiferin/nigricanside (xanthone) inhibited the bacterial extracellular communication of *V. campbellii* BAA1118 (see Table 5).

## 4. Discussion

Oral diseases, such as dental caries and periodontitis, are mostly linked with microbial biofilms growing in the form of supragingival and subgingival plaque. The development of oral biofilms has led to their persistence with conventional antimicrobial therapies. In view of the growing need for a new remedy for oral infection treatment, naturally occurring molecules found in the plant kingdom may become important candidates for the development of new bacterial biofilm inhibitors. This study assessed the ability of 15 methanol extracts of five different *Iris* plant species to modulate mono- and multi-species oral biofilms. Moreover, in this study, the extracts were further analyzed for potential phytochemical components using UHPLC-HRMS/MS-targeted screening, and some of the detected compounds may associate with the activity observed against the tested bacterial biofilms.

To the best of our knowledge, this is the first report on the phytochemical compounds in methanol extracts of the aforementioned plant species characterized by the UHPLC-HRMS/MS technique. The chemical profile of our *Iris* extracts is similar to what has been previously reported. In many *Iris* species, (iso)flavonoids exist as the main class of polyphenolics as published by [29] and in this study, where more than 90 compounds of this group were identified. Iristectorigenin A, irisflorentin, iriskumaon, and irilone were previously observed in *I. germanica* and *I. pallida* extracts [30], while Rahman et al. (2002, 2003) demonstrated the presence of tectorigenin, irisolidone, irigenin S, iridin, 5-hydroxy-4’-methoxy-6,7-methylenedioxyisoflavone, irilone 4’-O-β-d-glucopyranoside, irifloside, nigricin, and germanasim B in *I. germanica* rhizomes [31,32]. 4′-O-methylapigenin 6-C-hexoside, 4′-O-methylapigenin 8-C-hexoside, already identified in rhizomes of *I. pseudopumila* [33], were also tentatively confirmed in *I. germanica*. On the other hand, the identified terpenoid compounds in our extracts were quite different from those found in the same plant from other sources reported previously. To date, 21-desoxyiridogermanal, 21-desoxyiridogermanal, 26-hydroxyiridal, spirocyclic hemiacetal, and 17Ɛ, 26-dihydroxyiridal were not present in any of all five investigated *Iris* spp., while they were isolated and identified in other *Iris* spp. [34,35]. From these data, it appears that there is a species-specific variability in the phytochemical composition of *Iris* extracts.

The formation of bacterial biofilms plays a crucial role in the virulence of oral pathogenic strains and has become one of the major factors in the increasing emergence of antibiotic resistance. In this study, 15 methanolic extracts of *Iris* spp. were tested for their ability to inhibit planktonic cell adhesion to the surface and eradicate maturated biofilms. The obtained results for both mono- and multi-species oral biofilms highlight a dramatic reduction in bacterial biofilm formation on a polystyrene surface by extracts from *I. pallida, I. versicolor*, even at concentrations far below the MIC. The anti-adhesion activity of these extracts was observed in a concentration-dependent manner for both Gram-positive and Gram-negative bacteria during the initial stages of biofilm development. Furthermore, the application of higher concentrations was required to eliminate half of the bacterial cells attached in the form of a multi-species biofilm compared to the mono-species ones. The result of biofilm eradication demonstrated a reduced biomass of mature biofilm on the polystyrene surface by oral bacterial strains when treated with different concentrations of methanolic extracts of *I. pallida* and *I. versicolor*. The extract from *I. pallida* leaves exhibited the strongest anti-biofilm activity at an IC_50_ of 0.29 ± 0.01 g/L for disrupting a mature *P. aeruginosa* biofilm. The evaluated extracts contain a wide range of secondary metabolites which have been thoroughly investigated for their potential to modulate bacterial activities, including planktonic cell adherence, virulence, and differentiation. Quercetin, a flavonoid also detected in the studied *Iris* extracts, has been previously tested for its inhibition of biofilm development containing oral bacteria, *P. aeruginosa*, *S. aureus* strains, and clinically isolated MRSA strains [36,37,38,39]. Interestingly, flavonoids exhibited strong sortase inhibitory activity, which is an enzyme that is responsible for modulating the attachment ability of cells to host tissue and the production of surface protein virulence factors to the peptidoglycan cell wall layer of Gram-positive bacteria such as *Streptococcus mutans* and *Streptococcus pneumoniae* [38,40]. The inhibitory effect on the *S. aureus* sortase A activity of the dryocrassin ABBA flavonoid has been studied [41], and similar activity was also observed with the application of isovitexin at an IC_50_ of 28.98 mg/L [42]. Therefore, it is conceivable that the anti-biofilm potential of the extracts may be related to the ability of their phytochemical components to inactivate bacterial adhesins and enzymes altering the cell membrane, cell–substratum interactions, adherence phase, and biofilm maturation. Although the mechanism behind biofilm modulation is still unclear, the observed effects could result from a combination of multiple factors attributed to several mechanisms, such as interference cell–cell communication pathways such as the quorum sensing system [43].

The production of bioluminescence in *V. campellii* is positively regulated by a typical QS system responding to different autoinducers (AIs)-specifically, *N*-acyl homoserine lactones (AI-1) in Gram-negative bacteria, oligopeptides in Gram-positive bacteria, and a furanosyl borate diester or autoinducer-2 (AI-2) in both Gram-negative and Gram-positive bacteria [44]. As the bacterial QS-deficient mutant exhibits critical deficiencies in colonization and virulence, the modulation of QS systems can be considered an attractive approach to bacterial infection control [45]. Hence, the anti-QS potential of the *Iris* extracts used in our study was tested in terms of their ability to inhibit signal-based cell–cell communication. This activity was explored by using the standard strain of *V. campbellii* BAA1118 and BAA1119 responding to AI-1 and AI-2 autoinducer, respectively, as a biological model. The extracts from *I. lactea* and *I. carthaliniae* did not exhibit anti-QS activity at all, while the leaf extract from *I. pallida* significantly inhibited the bioluminescence production in both AI-1 and AI-2 systems similarly to the *I. versicolor* root and rhizome extracts. Despite the fact that the underlying mechanism of the extracts is not fully understood, our results suggest the involvement of the inhibition of AHL or interference with the cell–cell communication system in the anti-biofilm activity of the tested extracts.

In this work, we found that the *Iris* spp. methanol extracts, excluding the rhizome extracts from *I. versicolor* and *I. carthaliniae*, were not toxic to human fibroblast cells (MRC), suggesting that these extracts could be safely used as a therapeutic agent. 

Furthermore, a strong relationship between the biological activity of *Iris* extracts and their phytochemical compounds was confirmed using correlation analysis (Table 5). The ability of myristic acid to prevent the adherence of *Escherichia coli* planktonic cells suggested a potential of *Iris* extracts to inhibit biofilm formation [46], while based on the literature data, there have been no published studies to date that report on the anti-biofilm activity of germanaism. Myristic acid was shown to be a QS inhibitor, and it significantly inhibited the production of four extracellular virulence factors in the *P. aeruginosa* biofilm [47]. Prasath et al. reported the antibiofilm and antivirulence ability of myristic acid against *C. albicans* at a 125 mg/L concentration. The myristic acid-mediated regulation of the composition of lipid rafts may modulate the biofilm formation [48]. Our research also revealed that compounds of three different chemical classes: terpenoids, xanthones and steroids, inhibited QS in *V. campbellii* BAA1118.

Duckworth (2009) published a report about the sufficient effect of a two-minute use of mouthwash [49]. Therefore, the effectiveness of the substances within this short period on the modulation of both mono- and multi-species oral biofilms needs to be further evaluated, as well as the effectiveness of repeated exposures.

## 5. Conclusions

Natural products represent potential control agents to be used in therapeutic dental treatments. Here, we report on the phytochemical profile and the inhibitory effect on the growth and biofilm formation of mono- and multi-species oral biofilms of phytochemical-rich methanol extracts derived from selected *Iris* species, emphasizing their potency to modulate the virulence properties of dental plaque while maintaining oral health. Based on the highly heterogeneous data and the risk of bias, caution is required when interpreting the presented evidence. The *in vitro* mono- and multi-species biofilms used in our study clearly do not reflect the complex polymicrobial and environmental interactions present in the oral cavity. However, we found the extracts from *I. pallida* and *I. versicolor* in particular to both modulate biofilm formation with a higher effect on Gram-negative bacteria (*P. aeruginosa*), and also interfere with QS phenotype behaviors without affecting the growth of targeting bacteria as well as the human fibroblast cell lines (MRC). Therefore, it appears that *Iris* spp. is a potential candidate as an ecological caries-preventive agent that does not cause antibiotic tolerance and could be valuable in the field of dentistry and pharmacology for the production of oral care products. Further studies of controlled clinical trials with longer observation periods are required to identify multiple mechanisms of action, and efficacious and safe doses of the extracts.

## Figures and Tables

**Table 1:** Major chemical constituents present in assessed *Iris* extracts of leaves (L), roots (R), and rhizomes (Rh).

Species	Number of Detected Compounds (Tentative Identity)							
	(Iso)flavonoids	Phenols	Fatty Acids	Terpenoids	Steroids	Xanthones	Quinones	All
*I. pallida* (L)	35	5	8	14	4	5	2	73
*I. pallida* (R)	38	7	6	13	3	8	-	75
*I. pallida* (Rh)	15	11	6	8	3	9	-	52
*I. versicolor* (L)	8	7	7	20	3	13	-	58
*I. versicolor* (R)	11	10	7	17	4	9	-	58
*I. versicolor* (Rh)	5	10	7	22	3	10	-	57
*I. lactea* (L)	10	9	7	13	3	7	3	52
*I. lactea* (R)	12	11	5	10	4	7	1	50
*I. lactea* (Rh)	37	6	7	12	4	7	-	73
*I. carthaliniae* (L)	11	8	6	12	4	5	1	47
*I. carthaliniae* (R)	23	8	4	13	5	4	-	57
*I. carthaliniae* (Rh)	31	7	7	12	4	2	-	63
*I. germanica* (L)	19	5	9	17	4	6	3	63
*I. germanica* (R)	46	5	8	15	3	8	-	85
*I. germanica* (Rh)	43	7	5	10	4	9	-	78

L—leaves; R—roots; Rh—rhizomes.

**Table 2:** Concentration of *Iris* spp. extract halving respective activity: (1) adhesion of bacteria forming biofilm and (2) mature biofilm.

species	Anti-AdhesionIC_50_ [mg/L]			AntibiofilmIC_50_ [mg/L]		
	*S. aureus*	*P. aeruginosa*	Dental Plaque	*S. aureus*	*P. aeruginosa*	Dental Plaque
*I. pallida* (L)	139.4 ± 2.0	88.5 ± 1.6	163.5 ± 6.5	406.3 ± 20.4	287.0 ± 10.3	>666.7
*I. pallida* (R)	177.2 ± 13.0	122.7 ± 4.0	326.9 ± 13.5	>666.7	447.6 ± 45.9	>666.7
*I. pallida* (Rh)	334.5 ± 8.1	297.2 ± 23.1	556.6 ± 48.3	>666.7	628.2 ± 13.9	>666.7
*I. versicolor* (L)	169.0 ± 6.1	132.2 ± 14.8	357.0 ± 13.0	>666.7	497.2 ± 22.2	>666.7
*I. versicolor* (R)	161.0 ± 5.5	177.0 ± 4.3	315.3 ± 38.6	549.6 ± 8.3	526.1 ± 15.9	>666.7
*I. versicolor* (Rh)	98.5 ± 9.5	112.4 ± 5.2	201.6 ± 12.4	615.6 ± 28.9	500.4 ± 16.4	>666.7
*I. lactea* (L)	>666.7	>666.7	>666.7	>666.7	>666.7	>666.7
*I. lactea* (R)	255.3 ± 26.0	393.4 ± 17.0	542.8 ± 46.3	>666.7	>666.7	>666.7
*I. lactea* (Rh)	370.5 ± 22.6	>666.7	>666.7	>666.7	>666.7	>666.7
*I. carthaliniae* (L)	494.5 ± 69.1	512.8 ± 14.6	>666.7	>666.7	>666.7	>666.7
*I. carthaliniae* (R)	427.8 ± 19.1	>666.7	>666.7	>666.7	>666.7	>666.7
*I. carthaliniae* (Rh)	>666.7	>666.7	>666.7	>666.7	>666.7	>666.7
*I. germanica* (L)	178.1 ± 23.2	267.5 ± 8.6	357.4 ± 16.3	>666.7	513.0 ± 56.1	>666.7
*I. germanica* (R)	334.6 ± 8.4	>666.7	>666.7	>666.7	>666.7	>666.7
*I. germanica* (Rh)	258.1 ± 10.0	630.7 ± 19.0	542.0 ± 23.6	>666.7	>666.7	>666.7

L—leaves; R—roots; Rh—rhizomes; Data are presented as the average of 3 repetitions with SEM. For the statistical analysis, see the Supplementary Supplementary Table S2.

**Table 3:** Concentration of *Iris* spp. extract halving quorum sensing of *Vibrio campbellii*.

	*V. campbellii* BAA1118	*V. campbellii* BAA1119
**species**	QS IC_50_ [mg/L]	QS IC_50_ [mg/L]
*I. pallida* (L)	533.8 ± 3.2	605.0 ± 3.0
*I. pallida* (R)	560.6 ± 10.4	>666.7
*I. pallida* (Rh)	>666.7	>666.7
*I. versicolor* (L)	543.4 ± 59.5	>666.7
*I. versicolor* (R)	542.0 ± 1.8	644.0 ± 10.0
*I. versicolor* (Rh)	638.5 ± 16.2	597.2 ± 33.2
*I. lactea* (L)	>666.7	>666.7
*I. lactea* (R)	>666.7	>666.7
*I. lactea* (Rh)	>666.7	>666.7
*I. carthaliniae* (L)	>666.7	>666.7
*I. carthaliniae* (R)	>666.7	>666.7
*I. carthaliniae* (Rh)	>666.7	>666.7
*I. germanica* (L)	297.1 ± 10.3	>666.7
*I. germanica* (R)	>666.7	>666.7
*I. germanica* (Rh)	>666.7	>666.7
Erythromycin	20.7 ± 1.0	2.6 ± 0.1

L—leaves; R—roots; Rh—rhizomes. Data presented as average of 3 repetitions with SEM. For the statistical analysis, see Supplementary Table S2.

**Table 4:** Concentration of *Iris* spp. extract halving viability of fibroblasts (MRC) cell line.

*Iris* part	IC_50_ [mg/L]
*I. pallida* (L)	>666.7
*I. pallida* (R)	>666.7
*I. pallida* (Rh)	>666.7
*I. versicolor* (L)	>666.7
*I. versicolor* (R)	>666.7
*I. versicolor* (Rh)	96.0 ± 6.7
*I. lacteal* (L)	>666.7
*I. lacteal* (R)	>666.7
*I. lacteal* (Rh)	>666.7
*I. carthaliniae* (L)	>666.7
*I. carthaliniae* (R)	>666.7
*I. carthaliniae* (Rh)	317.3 ± 40.0
*I. germanica* (L)	>666.7
*I. germanica* (R)	>666.7
*I. germanica* (Rh)	>666.7
Doxorubicin	0.4 ± 0.007

L–leaves; R–roots; Rh–rhizomes. Data are presented as an average of 3 repetitions with SEM.

**Table 5:** Correlation coefficients (R^2^) of dependence of biological activity of 15 *Iris* extracts on HRMS/MS responses of compounds detected and tentatively identified by targeted UHPLC–HRMS/MS screening. Note: only substances for which the correlation was significant are presented.

Compound	Inhibition of Dental Biofilm Adhesion			Inhibition of BA1118 Communication		
	df	R_crit_	R^2^	df	R_crit_	R^2^
Myristic acid	7	0.666	0.753			
Germanaism B	1	0.997	0.999			
7-β-hydroxystigmast-4-en-3-one				4	0.811	0.933
Amorphene/α-muurolene/β-gurjuenene/γ-elemene				3	0.878	0.932
Isomangiferin/Mangiferin/Nigricanside				4	0.811	0.827

## Supplementary Materials

The following are available online at https://www.mdpi.com/2079-6382/9/7/403/s1, Table S1: Disruption of mature biofilm: one-way analysis of variance (ANOVA) followed by Duncan’s post hoc test (*p* < 0.05) to show significant differences between methanol extracts of *Iris* spp. at concentration of 666.7 mg/L, Table S2: Concentration of Iris spp. extract halving respective activity: (1) adhesion of bacteria forming biofilm and (2) mature biofilm: one-way analysis of variance (ANOVA) followed by Duncan’s post hoc test (*p* < 0.05) showing significant differences between methanol extracts of *Iris* spp. at concentration of 666.7 mg/L, Table S3: Concentration of *Iris* spp. extract halving quorum sensing of *Vibrio campbellii*: one-way analysis of variance (ANOVA) followed by Duncan’s post hoc test (*p* < 0.05) showing significant differences between methanol extracts of Iris spp. at concentration of 666.7 mg/L.

## Appendix A

**Table A1:** Overview of phytochemicals detected and tentatively identified in *Iris* spp. by ultra-high performance liquid chromatography-high-resolution tandem mass spectroscopy (UHPLC–HRMS/MS).

No.	Compound Name	Molecular Formula	Neutral Mass *	t_R_ (min)	*I. pallida*.			*I. versicolor*			*I. lactea*			*I. carthaliniae*			*I. germanica*		
					L	R	Rh	L	R	Rh	L	R	Rh	L	R	Rh	L	Rh	R
Xanthones																			
1	Isomangiferin/Mangiferin/Nigricanside	C_19_H_18_O_11_	422.0844	1.03	-	-	-	-	+	-	-	-	-	-	-	-	-	+	+
2	Iriflophenone	C_13_H_10_O_5_	246.0523	2.08	-	-	+	+	+	+	+	+	-	+	+	-	-	-	-
3	Iriflophenone 2-O-hexoside/Iriflophenone 4-O-hexoside	C_19_H_20_O_10_	408.1039	2.5	-	-	+	-	-	+	+	+	-	-	-	-	-	-	-
4	Iriflophenone	C_13_H_10_O_5_	246.0521	2.69	-	-	+	+	+	+	-	+	-	+	+	-	-	-	-
5	Iriflophenone	C_13_H_10_O_5_	246.0529	2.87	-	-	-	+	-	-	-	-	-	-	-	-	+	+	+
6	Iriflophenone 2-O-hexoside/Iriflophenone 4-O-hexoside	C_19_H_20_O_10_	408.1053	2.88	+	+	+	+	-	+	+	-	+	+	-	-	+	+	+
7	Isomangiferin/Mangiferin/Nigricanside	C_19_H_18_O_11_	422.0845	2.96	+	+	+	+	+	+	+	+	+	-	-	-	+	+	+
8	4-O-methyliriflophenone	C_14_H_12_O_5_	260.0685	3.09	-	+	-	-	-	-	-	-	+	-	-	-	-	-	+
9	7-O-methylisomangiferin/7-O-methylmangiferin	C_20_H_20_O_11_	436.0995	3.24	+	+	+	+	-	-	-	-	+	-	-	-	+	+	+
10	Iriflophenone	C_13_H_10_O_5_	246.0525	3.37	-	+	+	-	+	+	-	-	+	-	-	-	-	+	+
11	Isomangiferin/Mangiferin/Nigricanside	C_19_H_18_O_11_	422.0844	3.39	-	-	-	+	-	-	-	-	-	-	-	-	-	-	-
12	Bellidifolin	C_14_H_10_O_6_	274.047	3.45	-	-	-	+	-	-	-	-	-	-	-	-	-	-	-
13	4-O-methyliriflophenone	C_14_H_12_O_5_	260.0685	3.61	-	+	-	-	-	-	-	-	-	-	-	-	-	-	-
14	Bellidifolin	C_14_H_10_O_6_	274.047	3.62	-	-	-	+	+	+	+	-	-	-	-	+	-	-	-
15	7-O-methylisomangiferin/7-O-methylmangiferin	C_20_H_20_O_11_	436.0998	3.64	-	-	-	+	+	+	-	-	-	-	-	-	-	-	-
16	4-O-methyliriflophenone	C_14_H_12_O_5_	260.0674	3.91	+	+	+	+	+	+	+	+	+	+	+	+	+	+	+
17	Isomangiferin/Mangiferin/Nigricanside	C_19_H_18_O_11_	422.0836	6.52	-	-	-	+	-	-	-	-	-	-	-	-	-	-	-
18	Bellidifolin	C_14_H_10_O_6_	274.047	4.21	+	+	+	+	+	+	+	+	+	+	+	-	+	+	+
Phenols																			
19	Vanillic acid	C_8_H_8_O_4_	168.042	1.68	+	+	+	+	+	+	+	+	+	+	+	+	+	+	+
20	trans-Cinnamic acid	C_9_H_8_O_2_	148.0526	2.06	+	+	+	+	+	+	+	+	+	+	+	+	+	+	+
21	Salicylic acid/*p*-hydroxybenzoic acid	C_7_H_6_O_3_	138.0319	2.08	-	-	+	-	+	+	+	+	-	-	-	-	-	-	-
22	Protocatechuic acid	C_7_H_6_O_4_	154.0263	2.17	+	+	+	+	+	+	+	+	-	+	-	+	+	-	+
23	Ferulic acid	C_10_H_10_O_4_	194.0574	2.61	+	+	+	+	+	+	+	+	+	+	+	+	+	+	+
24	Salicylic acid/*p*-hydroxybenzoic acid	C_7_H_6_O_3_	138.0319	2.69	-	-	+	-	+	+	-	+	-	+	+	-	-	-	-
25	3-Hydroxy-5-acetophenone/3-Hydroxy-5-methoxyacetonphenoone/Apocynin	C_9_H_10_O_3_	166.0625	2.91	-	+	+	-	-	-	-	-	+	-	-	-	-	+	+
26	Salicylic acid/*p*-hydroxybenzoic acid	C_7_H_6_O_3_	138.0316	2.96	-	-	+	-	+	+	-	+	+	+	+	+	-	-	-
27	Caffeic acid	C_9_H_8_O_4_	180.042	2.96	+	-	-	+	+	-	+	+	-	-	+	+	-	-	-
28	Vanillic acid	C_8_H_8_O_4_	168.0419	2.98	-	+	+	+	+	+	+	+	+	+	+	+	-	+	+
29	Vanillic acid	C_8_H_8_O_4_	168.0419	3.5	-	-	+	+	-	+	+	+	-	-	-	-	-	-	-
30	Vanillic acid	C_8_H_8_O_4_	168.0419	3.75	-	+	+	-	+	+	+	+	-	+	+	-	+	-	+
Flavonoids and isoflavonoids																			
31	4’,7-di-O-methyldihydroquercetin-5,3,3’-trihydroxy-7,4’-dimethoxyflavanone	C_17_H_16_O_7_	332.0911	1.09	-	-	-	+	-	-	-	-	-	-	-	-	-	-	-
32	Luteolin 7-O-(2“-*p*- umaroyl) rhamnoside	C_30_H_26_O_12_	578.1409	2.71	-	-	-	-	+	+	+	+	-	+	+	-	-	-	-
33	Swertiajaponin/Tectoridin	C_22_H_22_O_11_	462.116	2.85	-	-	-	-	-	-	-	-	-	-	-	+	-	-	-
34	Apigenin 8-C-(2“-hexosyl) hexoside/Kaempferol 7-O-(6“-rhamnosyl) hexoside	C_27_H_30_O_15_	594.1577	2.85	+	-	-	-	-	-	+	-	-	-	-	+	+	-	-
35	Isorhamnetin 3-O-(2“-hamnosyl) hexoside/Isorhamnetin 3-O-(6“-rhamnosyl) hexoside/Tectorigenin-7-O-glucosyl-4′-O-glucoside	C_28_H_32_O_16_	624.169	2.85	-	-	-	-	-	-	-	-	-	-	-	+	-	-	-
36	Luteolin 7-O-(2“-*p*-oumaroyl)rhamnoside	C_30_H_26_O_12_	578.1413	2.88	-	-	-	-	-	-	-	-	-	+	+	-	-	-	-
37	Iristectorigenin A 7-O-hexuronide/Irisdichotin A/Iristectoridin B/Iristectorin A/Iristectorin B	C_23_H_24_O_12_	492.1255	2.9	-	-	-	-	-	-	-	-	-	-	-	+	-	-	-
38	Dichotomitin 3′-O-hexoside	C_24_H_24_O_12_	520.1204	2.92	-	-	-	+	-	-	-	-	-	-	-	-	-	-	-
39	Tectorigenin-4′-O-diglucoside/Tectorigenin-7-O-diglucoside	C_28_H_32_O_17_	640.1639	2.97	-	-	-	-	-	-	-	-	-	-	-	-	-	+	-
40	Iriflophenone 4-O-(6’’-acetyl) hexoside	C_21_H_22_O_11_	450.1154	3.03	-	-	+	-	-	+	-	+	-	-	-	+	-	-	-
41	Apigenin 8-C-(2“-pentosyl) hexoside/Isoschaftoside/Schaftoside	C_26_H_28_O_14_	564.1477	3.05	-	+	-	+	-	-	-	-	-	-	+	-	+	+	+
42	Kaempferol 3-O-galactoside/Isoorientin/Kaempferol 3-O-glucoside/Luteolin 6-C-glucoside/Luteolin 8-C-hexoside	C_21_H_20_O_11_	448.1	3.1	+	-	-	+	-	-	+	-	-	+	-	-	+	-	
43	Swertiajaponin/Tectoridin	C_22_H_22_O_11_	462.115	3.14	+	-	-	-	-	-	-	-	-	+	-	-	+	-	
44	7,4’-dimethoxy-8,3’,5’-trihydroxy-6-O-β-D-glucopyranosylisoflavone	C_23_H_24_O_13_	508.1207	3.16	+	+	-	-	-	-	-	-	+	-	-	-	-	-	+
45	Iridin	C_24_H_26_O_13_	522.1371	3.16	+	+	-	-	-	-	-	-	+	-	-	-	-	-	+
46	Tectorigenin-4′-O-diglucoside/Tectorigenin-7-O-diglucoside	C_28_H_32_O_17_	640.1629	3.17	-	-	-	-	-	-	-	-	-	-	-	+	-	-	-
47	Quercetin 3-O-galactoside/Quercetin 3-O-glucoside	C_21_H_20_O_12_	464.0946	3.18	-	+	-	-	-	-	-	-	-	-	-	-	+	-	+
48	Apigenin 6-C-hexoside/Apigenin 8-C-glucoside/Isovitexin	C_21_H_20_O_10_	432.1055	3.19	-	-	-	-	-	-	-	-	-	-	+	-	-	-	-
49	Apigenin 8-C-(2“-pentosyl) hexoside/Isoschaftoside/Schaftoside	C_26_H_28_O_14_	564.1475	3.19	-	-	-	-	-	-	-	-	-	-	+	-	-	-	-
50	Germanaism A/5,4′-Methoxy-6,7-methylenedioxy-isoflavone-3′-O-β-d-glucopyranoside	C_24_H_24_O_12_	504.1253	3.2	-	-	-	-	-	-	-	-	-	-	+	+	+	-	-
51	Isorhamnetin 3-O-(2“-hamnosyl) hexoside/Isorhamnetin 3-O-(6“-rhamnosyl) hexoside/Tectorigenin-7-O-glucosyl-4′-O-glucoside	C_28_H_32_O_16_	624.1692	3.2	-	-	-	-	-	-	-	-	-	-	+	+	-	+	+
52	Genistein	C_15_H_10_O_5_	270.0521	3.24	-	-	-	-	-	-	-	-	-	-	+	+	-	+	+
53	Swertiajaponin/Tectoridin	C_22_H_22_O_11_	462.1159	3.24	-	-	-	-	-	-	-	-	-	-	-	+	-	-	-
54	Iristectorigenin A 7-O-hexuronide/Irisdichotin A/Iristectoridin B/Iristectorin A/Iristectorin B	C_23_H_24_O_12_	492.1268	3.27	-	-	-	-	-	-	-	-	-	-	-	-	-	+	+
55	Apigenin 8-C-(2“-hexosyl) hexoside/Kaempferol 7-O-(6“-rhamnosyl) hexoside	C_27_H_30_O_15_	594.1579	3.27	-	-	-	-	-	-	-	-	-	-	-	+	-	+	+
56	Irilin D	C_16_H_12_O_7_	316.0571	3.29	-	-	+	-	+	-	+	+	-	-	+	+	-	-	-
57	Iristectorigenin B/Iristectorigenin A	C_17_H_14_O_7_	330.0739	3.29	-	+	-	-	-	-	+	+	+	-	-	+	-	+	+
58	6,3’,4’-trimethoxy-7,8,5’-trihydroxyisoflavone/Irigenin	C_18_H_16_O_8_	360.0834	3.32	+	+	-	-	-	-	-	-	+	-	-	-	-	-	-
59	Apigenin 6-C-hexoside/Apigenin 8-C-glucoside/Isovitexin	C_21_H_20_O_10_	432.1043	3.32	-	-	-	-	-	-	+	-	-	-	-	-	+	-	-
60	Genistein	C_15_H_10_O_5_	270.0533	3.33	+	+	-	-	-	-	-	-	+	-	+	+	-	+	+
61	4’,7-di-O-methyldihydroquercetin-5,3,3’-trihydroxy-7,4’-dimethoxyflavanone	C_17_H_16_O_7_	332.0895	3.35	-	-	-	-	-	-	-	-	-	-	-	-	-	+	+
62	Quercetin 3-O-galactoside/Quercetin 3-O-glucoside	C_21_H_20_O_12_	464.0948	3.35	-	+	-	-	-	-	-	-	-	-	-	-	+	-	+
63	Apigenin 8-C-(2“-pentosyl) hexoside/Isoschaftoside/Schaftoside	C_26_H_28_O_14_	564.1467	3.35	+	-	-	-	-	-	-	-	+	-	-	+	-	-	-
64	Irilin A/Tectorigenin	C_16_H_12_O_6_	300.0633	3.37	+	+	-	-	-	-	-	-	+	-	+	+	-	+	+
65	4′-O-Methylapigenin 6-C-hexoside/4′-O-Methylapigenin 8-C-hexoside/Swertisin	C_22_H_22_O_10_	446.1214	3.37	-	-	-	-	-	-	-	-	-	-	-	-	+	-	-
66	3′-Hydroxyltectoridin/Isorhamnetin 3-O-galactoside/Isorhamnetin 3-O-glucoside	C_22_H_22_O_12_	478.11	3.43	-	-	-	+	-	-	-	-	-	-	-	-	-	-	-
67	Iristectorigenin B/Iristectorigenin A	C_17_H_14_O_7_	330.0731	3.44	+	+	-	-	-	-	-	-	+	-	-	+	-	+	+
68	Iristectorigenin A 7-O-hexuronide/Irisdichotin A/Iristectoridin B/Iristectorin A/Iristectorin B	C_23_H_24_O_12_	492.1257	3.45	-	+	-	-	-	-	-	-	+	-	-	+	-	+	+
69	Germanaism B	C_23_H_22_O_11_	474.1161	3.47	+	+	-	-	-	-	-	-	+	-	-	-	-	+	+
70	Swertiajaponin/Tectoridin	C_22_H_22_O_11_	462.1155	3.48	+	+	+	-	-	-	-	-	+	-	-	-	-	+	+
71	Apigenin-6,8-di-C-arabinoside	C_25_H_26_O_13_	534.1363	3.48	-	-	-	-	-	-	-	-	-	+	-	-	-	-	-
72	Apigenin 8-C-(2“-hexosyl)hexoside/Kaempferol 7-O-(6“-rhamnosyl)hexoside	C_27_H_30_O_15_	594.1575	3.48	-	-	-	-	-	-	-	-	-	+	-	-	-	-	-
73	6,3’,4’-trimethoxy-7,8,5’-trihydroxyisoflavone/Irigenin	C_18_H_16_O_8_	360.0839	3.49	+	+	+	-	-	-	-	-	+	-	-	-	+	+	+
74	Iridin	C_24_H_26_O_13_	522.1378	3.5	+	+	-	-	-	-	-	-	+	-	-	-	+	+	+
75	Irilin D	C_16_H_12_O_7_	316.0583	3.56	-	-	-	-	-	-	-	-	-	-	-	+	-	-	-
76	Irilin A/Tectorigenin	C_16_H_12_O_6_	300.0634	3.58	-	-	-	-	-	-	-	-	-	-	-	+	-	-	-
77	Irilone A	C_16_H_10_O_6_	298.0478	3.6	+	+	-	-	-	-	-	-	+	-	-	-	-	+	+
78	Irilone 4′-O-hexoside/Irilone 4′-O-β-d-glucopyranoside/Irilone B	C_22_H_20_O_11_	460.1007	3.6	+	+	-	-	-	-	-	-	+	-	-	-	-	+	+
79	5,3’,4’,5’-tetramethoxy-6,7-methylenedioxyisoflavone	C_20_H_18_O_9_	402.0943	3.62	-	-	-	-	-	-	-	-	-	-	-	+	-	-	-
80	Swertiajaponin/Tectoridin	C_22_H_22_O_11_	462.1161	3.62	-	-	-	-	-	-	-	-	-	-	-	+	-	-	-
81	Irilone 4′-O-(6“-hexosyl)hexoside/Irilone 4′-O-β-d-glucopyranoside-(2 → 1)-l-rhamnoside	C_28_H_30_O_16_	622.1529	3.62	+	+	-	-	-	-	-	-	+	-	-	-	-	+	+
82	Germanaism B	C_23_H_22_O_11_	474.1167	3.67	+	+	-	-	-	-	-	-	+	-	-	-	-	+	+
83	Germanaism A/5,4′-Methoxy-6,7-methylenedioxy-isoflavone-3′-O-β-d-glucopyranoside	C_24_H_24_O_12_	504.1251	3.68	+	+	-	-	-	-	-	-	-	-	-	-	-	+	+
84	Apigenin-6,8-di-C-arabinoside	C_25_H_26_O_13_	534.1365	3.68	+	+	-	-	-	-	-	-	+	-	-	-	-	+	+
85	Apigenin 8-C-(2“-pentosyl) hexoside/Isoschaftoside/Schaftoside	C_26_H_28_O_14_	564.1444	3.68	+	+	-	-	-	-	-	-	+	-	-	-	-	+	+
86	4′-O-Methylapigenin 6-C-hexoside/4′-O-Methylapigenin 8-C-hexoside/Swertisin	C_22_H_22_O_10_	446.12	3.74	-	-	-	-	-	-	-	-	-	-	-	-	-	+	-
87	4’,7-di-O-methyldihydroquercetin-5,3,3’-trihydroxy-7,4’-dimethoxyflavanone	C_17_H_16_O_7_	332.0894	3.77	+	+	-	-	-	-	-	-	+	-	-	-	-	+	+
88	Irigenin S	C_19_H_18_O_8_	374.0989	3.78	+	+	-	-	-	-	-	-	+	-	-	-	-	+	+
89	Irilin B/Irisolidone	C_17_H_14_O_6_	314.0782	3.8	-	-	-	-	-	-	-	-	+	-	-	-	-	+	-
90	Embinin	C_29_H_34_O_14_	606.1955	3.81	+	-	-	-	-	-	-	-	-	-	-	-	-	-	-
91	4’-methyltectorigenin-7-glucoside/6,4’-dimethoxy-5-hydroxyflavone-7-glucoside/Irisdichotin C/Irisolidone 7-O-hexoside/Irisolidone-7-O-α-d-glucoside	C_23_H_24_O_11_	476.1302	3.82	-	-	-	-	-	-	-	-	-	-	-	-	-	+	-
92	Irilone 4′-O-[6“-(3-hydroxy-3-methylglutaryl)] hexoside	C_28_H_28_O_15_	604.1424	3.82	-	+	-	-	-	-	-	-	+	-	-	-	-	-	+
93	7-O-Methyltectorigenin 4′-O-(6“-hexosyl) hexoside	C_29_H_34_O_16_	638.1854	3.84	-	-	-	-	-	-	-	-	-	-	-	-	-	+	-
94	Irilone A	C_16_H_10_O_6_	298.0476	3.87	+	+	-	-	-	-	-	-	+	-	-	-	+	+	+
95	Irifloside/Irisflogenin-4′-O-[ß-d-glucopyranosyl(1 → 6)ß-d-glucopyranoside]	C_23_H_22_O_12_	490.1096	3.87	+	-	-	-	-	-	-	-	+	-	-	-	+	+	+
96	Irilone 4′-O-hexoside/Irilone 4′-O-β-d-glucopyranoside/Irilone B	C_22_H_20_O_11_	460.1006	3.89	+	+	-	-	-	-	-	-	+	-	-	-	-	+	+
97	4’-methyltectorigenin-7-glucoside/6,4’-dimethoxy-5-hydroxyflavone-7-glucoside/Irisdichotin C/Irisolidone 7-O-hexoside/Irisolidone-7-O-α-d-glucoside	C_23_H_24_O_11_	476.1315	4.02	-	-	+	-	-	-	-	+	-	-	-	-	-	+	-
98	Irilin D	C_16_H_12_O_7_	316.0577	4.07	-	-	+	-	+	-	+	+	-	+	+	+	-	-	-
99	4’-methyltectorigenin-7-glucoside/6,4’-dimethoxy-5-hydroxyflavone-7-glucoside/Irisdichotin C/Irisolidone 7-O-hexoside/Irisolidone-7-O-α-d-glucoside	C_23_H_24_O_11_	476.1307	4.13	-	-	-	-	-	-	-	-	-	-	-	-	-	+	-
100	Irilin B/Irisolidone	C_17_H_14_O_6_	314.0783	4.15	-	-	-	-	-	-	-	-	-	-	-	-	-	+	-
101	6,3’,4’-trimethoxy-7,8,5’-trihydroxyisoflavone/Irigenin	C_18_H_16_O_8_	360.084	4.15	+	+	+	-	-	-	-	-	+	-	-	-	+	+	+
102	Iristectorigenin B/Iristectorigenin A	C_17_H_14_O_7_	330.0731	4.18	+	+	+	-	+	-	-	+	+	-	-	+	+	+	+
103	Irisoid B/Irisoid C/Iriskashmirianin/4’,5-dimethoxy-3-hydroxy-6,7-methylenedioxyisoflavone/Nigricanin	C_18_H_14_O_7_	342.0737	4.25	-	+	-	-	-	-	-	-	-	-	+	-	-	+	+
104	Iristectorigenin B/Iristectorigenin A	C_17_H_14_O_7_	330.0728	4.27	-	-	-	-	-	-	-	-	-	+	-	-	-	-	-
105	Genistein	C_15_H_10_O_5_	270.0531	4.28	+	+	-	-	+	-	-	-	+	-	+	-	-	+	+
106	5-methoxy-4’-hydroxy-6,7-methylenedioxyisoflavone/Dichotomin/Nigricin	C_17_H_12_O_6_	312.0637	4.28	+	+	-	-	-	-	-	-	+	-	+	-	-	+	+
107	Irilin D	C_16_H_12_O_7_	316.0582	4.3	-	-	-	-	-	-	-	-	-	-	+	+	-	-	-
108	Iristectorigenin B/Iristectorigenin A	C_17_H_14_O_7_	330.0733	4.4	-	-	-	-	-	-	-	-	-	-	+	+	-	-	-
109	Irilin A/Tectorigenin	C_16_H_12_O_6_	300.0627	4.53	-	-	+	+	+	-	+	+	-	+	+	+	-	-	-
110	Irisflorentin a/Iriskumaonin methyl ether	C_19_H_16_O_7_	356.0893	4.53	-	+	+	-	-	-	-	-	+	-	-	-	+	-	+
111	Irigenin S	C_19_H_18_O_8_	374.099	4.54	+	+	-	-	-	-	-	-	+	-	-	-	-	+	+
112	Irisflorentin b	C_20_H_18_O_8_	386.1001	4.55	-	+	+	-	-	-	-	-	+	-	-	-	+	+	+
113	Irilin B/Irisolidone	C_17_H_14_O_6_	314.0778	4.57	+	+	-	-	-	+	-	-	+	-	-	-	-	+	+
114	dichotomitin	C_18_H_14_O_8_	358.0694	4.58	+	+	-	-	-	-	-	-	+	-	-	-	+	+	+
115	Irilone A	C_16_H_10_O_6_	298.0468	4.71	+	+	+	+	+	+	-	-	+	+	+	+	+	+	+
116	Irilin B/Irisolidone	C_17_H_14_O_6_	314.079	4.71	-	-	-	-	+	-	-	-	-	+	-	-	-	-	-
117	Irilin B/Irisolidone	C_17_H_14_O_6_	314.0792	4.86	-	-	-	-	-	-	-	-	-	-	+	+	-	-	-
118	Iristectorigenin B/Iristectorigenin A	C_17_H_14_O_7_	330.0732	4.88	-	-	-	-	-	-	-	-	-	-	+	+	-	-	-
119	Irilin B/Irisolidone	C_17_H_14_O_6_	314.078	5.01	-	-	+	-	-	-	+	+	-	-	+	+	-	-	-
120	Irilin B/Irisolidone	C_17_H_14_O_6_	314.0784	5.15	-	-	-	-	+	-	-	-	-	-	-	-	-	+	-
121	Irilin B/Irisolidone	C_17_H_14_O_6_	314.0782	5.41	-	-	+	-	-	-	-	+	-	-	+	-	-	-	-
122	Genistein	C_15_H_10_O_5_	270.0519	5.52	-	-	-	-	+	-	-	+	-	-	-	-	-	-	-
123	Irilone A	C_16_H_10_O_6_	298.0471	5.98	-	-	+	+	+	+	+	+	+	-	-	-	-	-	-
Terpenoids																			
124	Camphor	C_10_H_16_O	152.1202	3.87	-	+	-	-	+	-	-	-	-	-	+	-	-	-	+
125	22,23-epoxy-21-hydroxyiridal/Iritectol A/Iritectol B	C_30_H_50_O_5_	490.3656	5.59	-	-	-	+	-	+	-	-	-	-	-	+	+	+	-
126	22,23-epoxy-10-deoxy-21-hydroxyiridal/22,23-epoxyiridal/Isoiridogermanal	C_30_H_50_O_4_	474.3694	6.28	-	-	-	+	+	+	-	-	-	-	-	-	+	+	-
127	α-Dehydroirigermanal	C_30_H_48_O_3_	456.3604	6.29	-	-	-	-	+	+	-	-	-	-	-	-	+	+	-
128	α-Dehydroirigermanal	C_30_H_48_O_3_	456.3606	6.51	+	+	+	+	+	+	+	+	+	+	+	+	+	-	+
129	22,23-epoxy-10-deoxy-21-hydroxyiridal/22,23-epoxyiridal/Isoiridogermanal	C_30_H_50_O_4_	474.3692	6.51	+	+	+	+	+	+	+	+	+	+	+	+	+	-	+
130	spirobicyclic triterpenoid	C_30_H_48_O_5_	488.3487	6.54	-	-	-	-	-	-	-	-	-	-	-	+	-	-	-
131	22,23-epoxy-10-deoxy-21-hydroxyiridal/22,23-epoxyiridal/Isoiridogermanal	C_30_H_50_O_4_	474.3702	6.65	-	-	-	+	-	+	-	-	-	-	-	-	+	+	-
132	Belamcandal	C_32_H_48_O_6_	528.3444	6.72	+	+	-	-	+	-	+	+	-	+	+	-	+	-	-
133	α-Dehydroirigermanal	C_30_H_48_O_3_	456.3604	6.91	-	-	-	+	-	+	-	-	-	-	-	-	-	-	-
134	22,23-epoxy-10-deoxy-21-hydroxyiridal/22,23-epoxyiridal/Isoiridogermanal	C_30_H_50_O_4_	474.3706	6.92	-	-	-	+	-	+	-	-	-	-	-	-	-	-	-
135	Iridotectoral A/Iridotectoral B	C_30_H_46_O_5_	486.3333	6.96	+	+	+	+	+	+	+	+	+	+	+	+	+	+	-
136	Belamcandal	C_32_H_48_O_6_	528.3434	6.98	+	+	-	-	+	-	+	+	+	+	+	-	+	-	-
137	Belamcandal	C_32_H_48_O_6_	528.344	7.27	+	+	+	+	+	+	+	+	+	+	+	+	+	-	+
138	α-Dehydroirigermanal	C_30_H_48_O_3_	456.3601	7.34	-	+	-	-	-	+	-	-	+	-	-	-	-	-	+
139	spirobicyclic triterpenoid	C_30_H_48_O_5_	488.3486	7.51	-	-	+	+	+	+	+	-	-	-	-	-	-	-	-
140	Iridal/α-irigermanal	C_30_H_50_O_4_	458.3739	7.52	-	+	-	+	+	+	-	-	+	-	-	-	-	+	+
141	Iridotectoral A/Iridotectoral B	C_30_H_46_O_5_	486.3332	7.56	-	-	-	+	-	+	-	-	-	+	+	+	-	-	-
142	Iridotectoral A/Iridotectoral B	C_30_H_46_O_5_	486.3336	7.94	-	-	+	+	+	+	+	-	-	-	-	-	-	-	-
143	22,23-epoxy-10-deoxy-21-hydroxyiridal/22,23-epoxyiridal/Isoiridogermanal	C_30_H_46_O_5_	474.3696	7.97	-	-	-	+	-	+	-	-	-	-	-	-	-	+	-
144	Amorphene/α-Muurolene/β-Gurjuenene/γ-Elemene	C_15_H_24_	204.1866	8.57	+	+	-	+	+	-	+	-	+	+	-	-	+	+	+
145	α-Dehydroirigermanal	C_30_H_48_O_3_	456.36	8.86	-	-	-	+	-	+	-	-	-	-	-	-	-	+	-
146	Amorphene/α-Muurolene/β-Gurjuenene/γ-Elemene	C_15_H_24_	204.1877	9.26	-	-	-	+	-	+	-	-	-	-	-	+	-	-	-
147	Iridal/α-irigermanal	C_30_H_50_O_3_	458.3756	9.26	+	-	+	+	+	+	+	+	+	+	+	+	-	-	+
148	iriversical	C_31_H_52_O_3_	472.3899	9.75	+	-	-	-	-	-	-	-	-	-	+	+	+	-	-
149	iriversical	C_31_H_52_O_3_	472.3898	10.03	-	-	-	-	-	-	-	-	+	-	-	-	-	+	-
150	Palmitic acid	C_16_H_32_O_2_	256.2397	10.7	+	+	+	+	+	+	+	+	+	+	+	+	+	+	+
151	Iridotectoral A/Iridotectoral B	C_30_H_46_O_5_	486.3347	10.9	-	+	-	-	+	-	-	+	-	-	+	-	-	-	-
152	Aristolone	C_15_H_22_O	218.1669	11.02	+	-	-	-	-	-	-	-	-	-	-	+	+	+	-
153	α-Dehydroirigermanal	C_30_H_48_O_3_	456.359	11.29	-	-	-	-	+	+	-	-	-	-	-	-	-	-	-
154	α-Dehydroirigermanal	C_30_H_48_O_3_	456.3594	11.49	-	+	-	-	-	+	-	+	-	-	+	+	-	+	+
155	Iridial	C_29_H_48_O_3_	444.3602	12.19	+	-	-	+	-	-	+	-	-	+	-	-	+	-	-
156	iriversical	C_31_H_52_O_3_	472.3921	13.5	-	-	-	-	-	+	-	-	-	-	-	-	-	-	-
157	Irisgermanic C	C_28_H_48_O_3_	432.36	13.55	+	-	-	-	-	-	+	-	-	-	-	-	+	-	-
158	Iridial	C_29_H_48_O_3_	444.3599	13.55	+	-	-	-	-	-	-	-	-	+	-	-	+	-	-
159	22,23-epoxy-10-deoxy-21-hydroxyiridal/22,23-epoxyiridal/Isoiridogermanal	C_30_H_50_O_4_	474.3693	13.46	-	+	-	-	+	-	-	+	-	-	-	-	-	+	+
160	Iridal/α-irigermanal	C_30_H_50_O_3_	458.3731	13.62	+	-	+	+	-	-	+	-	-	+	-	-	+	-	-
161	Amorphene/α-Muurolene/β-Gurjuenene/γ-Elemene	C_15_H_24_	204.19	14.01	-	-	-	-	-	-	-	-	+	-	-	-	-	+	-
Fatty acids																			
162	Myristic acid	C_14_H_28_O_2_	228.2078	4.98	+	-	-	-	-	-	+	-	-	+	-	-	+	-	-
163	Lauric acid	C_12_H_24_O_2_	200.1766	6.23	-	+	-	-	-	-	-	-	-	-	-	-	-	-	-
164	Myristic acid	C_14_H_28_O_2_	228.2079	7.92	+	+	-	-	-	-	-	-	+	-	-	-	+	+	+
165	Lauric acid	C_12_H_24_O_2_	200.1771	8.08	+	+	+	+	+	+	+	+	+	+	+	+	+	+	+
166	Myristic acid	C_14_H_28_O_2_	228.2084	9.36	+	+	+	+	+	+	+	+	+	+	+	+	+	+	+
167	Lauric acid	C_12_H_24_O_2_	200.1769	11.28	-	-	-	+	+	+	-	+	+	-	-	-	+	+	-
168	Stearic acid	C_18_H_36_O_2_	284.2706	11.87	+	+	+	+	+	+	+	+	+	+	+	+	+	+	+
169	Myristic acid	C_14_H_28_O_2_	228.2081	12.13	+	-	-	-	-	-	-	-	-	-	-	-	+	+	-
170	Myristic acid	C_14_H_28_O_2_	228.208	12.38	+	-	-	-	-	-	-	-	+	-	-	+	+	+	-
171	Palmitic acid	C_16_H_32_O_2_	256.2392	13.46	-	-	+	+	+	+	+	-	-	-	-	+	-	-	-
172	Stearic acid	C_18_H_36_O_2_	284.2704	13.76	-	-	+	+	+	+	+	-	-	+	-	+	-	-	-
Steroids																			
173	Stigmasterol	C_29_H_48_O	412.3688	9.73	-	-	-	-	-	-	-	-	-	-	+	+	-	-	-
174	Stigmasterol-3-O-b-D-glucopyranoside	C_35_H_58_O_6_	574.422	11.91	+	+	+	+	+	+	+	+	+	+	+	+	+	+	+
175	Stigmasterol-3-O-b-D-glucopyranoside	C_35_H_58_O_6_	574.4219	12.32	+	+	+	+	+	+	+	+	+	+	+	+	+	+	+
176	7-β-Hydroxystigmast-4-en-3-one	C_29_H_48_O_2_	428.3655	12.65	+	+	+	+	+	+	+	+	+	+	+	+	+	+	+
177	Stigmasterol	C_35_H_58_O_6_	412.3701	13.48	+	+	-	-	+	-	-	+	+	+	+	-	+	-	+
Quinones																			
178	Irisoquin B	C_24_H_40_O_4_	392.294	8.4	-	-	-	-	-	-	+	+	-	+	-	-	+	-	-
179	Irisquinone A	C_24_H_38_O_3_	374.282	11.32	+	-	-	-	-	-	+	-	-	-	-	-	+	-	-
180	Pallasone B Dihydroirisquinone	C_24_H_40_O_3_	376.2976	12.26	+	-	-	-	-	-	+	-	-	-	-	-	+	-	-

t_R_ (min)—retention time; L—leaves; R—roots; Rh—rhizomes; + standards for detected and - standards for not detected compound. * Experimentally obtained neutral exact mass.
