# Supplementary material for: Phytochemical Composition and In Vitro Biological Activity of Iris spp. (Iridaceae): A New Source of Bioactive Constituents for the Inhibition of Oral Bacterial Biofilms
Source: Antibiotics (Basel). 2020 Jul 11;9(7):403. doi: 10.3390/antibiotics9070403 (PMC7399867; doi:10.3390/antibiotics9070403)
Supplement: Supplementary file 1 [file antibiotics-09-00403-s001.pdf]

Article

Phytochemical Composition and *In Vitro* Biological Activity of *Iris* spp. (Iridaceae): A New Source of Bioactive Constituents for the Inhibition of Oral Bacterial Biofilms

Lan Hoang [1], František Beneš [2], Marie Fenclová [2], Olga Kronusová [1,3], Viviana Švarcová [1], Kateřina Řehořová [1], Eva Baldassarre Švecová [4], Miroslav Vosátka [4], Jana Hajšlová [2], Petr Kaštánek [3], Jitka Viktorová [1,*] and Tomáš Ruml [1]

[1] Department of Biochemistry and Microbiology UCT Prague, Faculty of Food and Biochemical Technology, Technická 3, 166 28 Prague, Czech Republic; hoangl@vscht.cz (L.H.); kronusova@ecofuel.cz (O.K.); fuchsovv@vscht.cz (V.S.); rehorova@vscht.cz (K.R.); prokesoj@vscht.cz (J.V.); rumlt@vscht.cz (T.R.)

[2] Department of Food Analysis and Nutrition UCT Prague, Faculty of Food and Biochemical Technology, Technická 3, 166 28 Prague, Czech Republic; benesfr@vscht.cz (F.B.); fenclovm@vscht.cz (M.F.); hajslovj@vscht.cz (J.H.)

[3] EcoFuel Laboratories Ltd., Ocelářská 9, 190 00 Praha, Czech Republic; kronusova@ecofuel.cz (O.K.); kastanek@ecofuel.cz (P.K.)

[4] Institute of Botany of the Czech Academy of Sciences, Zámek 1, 252 43 Průhonice, Czech Republic; eva.svecova@ibot.cas.cz (E.B.S.); miroslav.vosatka@ibot.cas.cz(M.V.)

* Correspondence: prokesoj@vscht.cz

Received: 16 June 2020; Accepted: 8 July 2020; Published: date

Supplementary Table 1. Disruption of mature biofilm: one-way analysis of variance (ANOVA) followed by Duncan´s post hoc test ($P<0.05$) to show significant differences between methanol extracts of Iris spp. at concentration of 666.7 mg/L. Different letters (e.g., a and b) indicate significant differences based on post hoc Duncan´s test ($p \leq 0.05$). Similar or shared letters (e.g., a and ab) indicate no significant differences ($p \leq 0.05$).

	S. aureus	*P. aeruginosa*	Dental plaque
I. pallida (L)	ab	a	jkl
I. pallida (R)	efghi	b	efghij
I. pallida (Rh)	hij	b	fghij
I. versicolor (L)	ijk	b	ghij
I. versicolor (R)	b	b	ghij
I. versicolor (Rh)	b	b	hij
I. lactea (L)	pgr	klm	qr
I. lactea (R)	c	defgh	klm
I. lactea (Rh)	pqr	lmnop	pqr
I. carthaliniae (L)	lmno	def	qr
I. carthaliniae (R)	jkl	lmn	qr
I. carthaliniae (Rh)	mnop	lmnop	opq
I. germanica (L)	cd	b	fghij
I. germanica (R)	nopq	rs	opq
I. germanica (Rh)	cd	cde	defg
Non-treated biofilm	s	s	s

L – leaves; R – roots; Rh – rhizomes.

Supplementary Table 2. Concentration of Iris spp. extract halving respective activity: (1) adhesion of bacteria forming biofilm and (2) mature biofilm*:* one-way analysis of variance (ANOVA) followed by Duncan´s post hoc test ($P < 0.05$) showing significant differences between methanol extracts of *Iris* spp. at concentration of 666.7 m*g/L*. Different letters (e.g., a and b) indicate significant differences

based on post hoc Duncan´s test ($P \leq 0.05$). Similar or shared letters (e.g., a and ab) indicate no significant differences ($P \leq 0.05$).

	Anti-adhesion IC_{50} [mg/L]			Anti-biofilm IC_{50} [mg/L]	
	S. aureus	*P. aeruginosa*	Dental plaque	*S. aureus*	*P. aeruginosa*
I. pallida (L)	ab	a	a	a	a
I. pallida (R)	b	ab	b		b
I. pallida (Rh)	d	d	c		c
I. versicolor (L)	ab	b	b		b
I. versicolor (R)	ab	c	b	b	b
I. versicolor (Rh)	a	ab	a	b	b
I. lactea (L)					
I. lactea (R)	c	e	c		
I. lactea (Rh)	de				
I. carthaliniae (L)	f	f			
I. carthaliniae (R)	ef				
I. carthaliniae (Rh)					
I. germanica (L)	b	d	b		b
I. germanica (R)	d				
I. germanica (Rh)	c	g	c		

L – leaves; R – roots; Rh – rhizomes.

Supplementary Table 3. Concentration of *Iris* spp. extract halving quorum sensing of *Vibrio campbellii*: one-way analysis of variance (ANOVA) followed by Duncan´s post hoc test ($P<0.05$) showing significant differences between methanol extracts of *Iris* spp. at concentration of 666.7 m*g/L*. Different letters (e.g. a and b) indicate significant differences based on post-hoc Duncan´s test ($p \leq 0.05$). Similar or shared letters (e.g. a and ab) indicate no significant differences ($p \leq 0.05$).

	***V. campbellii* BAA1118**	***V. campbellii* BAA1119**
	QS IC_{50} [mg/L]	QS IC_{50} [mg/L]
I. pallida (L)	c	b
I. pallida (R)	cd	
I. pallida (Rh)		
I. versicolor (L)	c	
I. versicolor (R)	c	b
I. versicolor (Rh)	d	b
I. lactea (L)		
I. lactea (R)		
I. lactea (Rh)		
I. carthaliniae (L)		
I. carthaliniae (R)		
I. carthaliniae (Rh)		
I. germanica (L)	b	
I. germanica (R)		
I. germanica (Rh)		
Erythromycin	a	a

L – leaves; R – roots; Rh – rhizomes.
